# Evaluation of combined growth media for in vitro cultivation of oropharyngeal biofilms on prosthetic silicone

**DOI:** 10.1007/s10856-018-6051-7

**Published:** 2018-04-09

**Authors:** M. Leonhard, B. Zatorska, D. Moser, Y. Tan, B. Schneider-Stickler

**Affiliations:** 1 Department of Otorhinolaryngology, Medical University of Vienna, Waehringer Guertel 18-20, 1090 Vienna, Austria; 2 Department of Cranio-Maxillofacial and Oral Surgery, Medical University of Vienna, Waehringer Guertel 18-20, 1090 Vienna, Austria

## Abstract

In the upper aerodigestive tract, biofilm deposits by oropharyngeal microbes can cause failure of medical polymer devices like voice prostheses. Previous studies on testing of inhibitive strategies still lack of comparability due to varying study protocols concerning growth media, microbial species and growth conditions. Goal of the study was therefore to test cultivation of a mixed biofilm of isolated oropharyngeal microbes under in vitro growth conditions using mixtures of common growth media. Mixtures of yeast peptone dextrose medium (YPD), fetal bovine serum (FBS), RPMI 1640, Yeast nitrogen base medium (YNB) and brain heart infusion (BHI) were tested to grow mixed biofilm deposits of Candida albicans, Candida tropicalis, Staphylococcus aureus, Streptococcus epidermidis, Rothia dentocariosa and Lactobacillus gasseri on medical grade silicone. Periodic assessment of living biofilm was performed over 22 days by a digital microscope and the cultivated biofilm structures were analyzed by scanning electron microscopy after completion of the study. Mixtures of BHI, YPD and FBS improved microscopic growth of multispecies biofilm deposits over time, while addition of RPMI and YNB resulted in reduction of visible biofilm deposit sizes. A mixtures of FBS 30% + YPD 70% and BHI 30% + YPD 70% showed enhanced support of permanent surface growth on silicone. Growth kinetics of in vitro multispecies biofilms can be manipulated by using mixtures of common growth media. Using mixtures of growth media can improve growth of longterm multispecies oropharyngeal biofilm models used for in vitro testing of antibiofilm materials or coatings for voice prostheses.

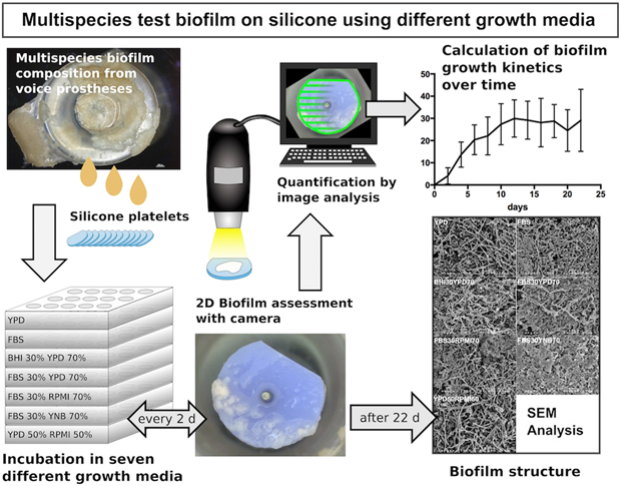

## Introduction

**Fig. 1:**
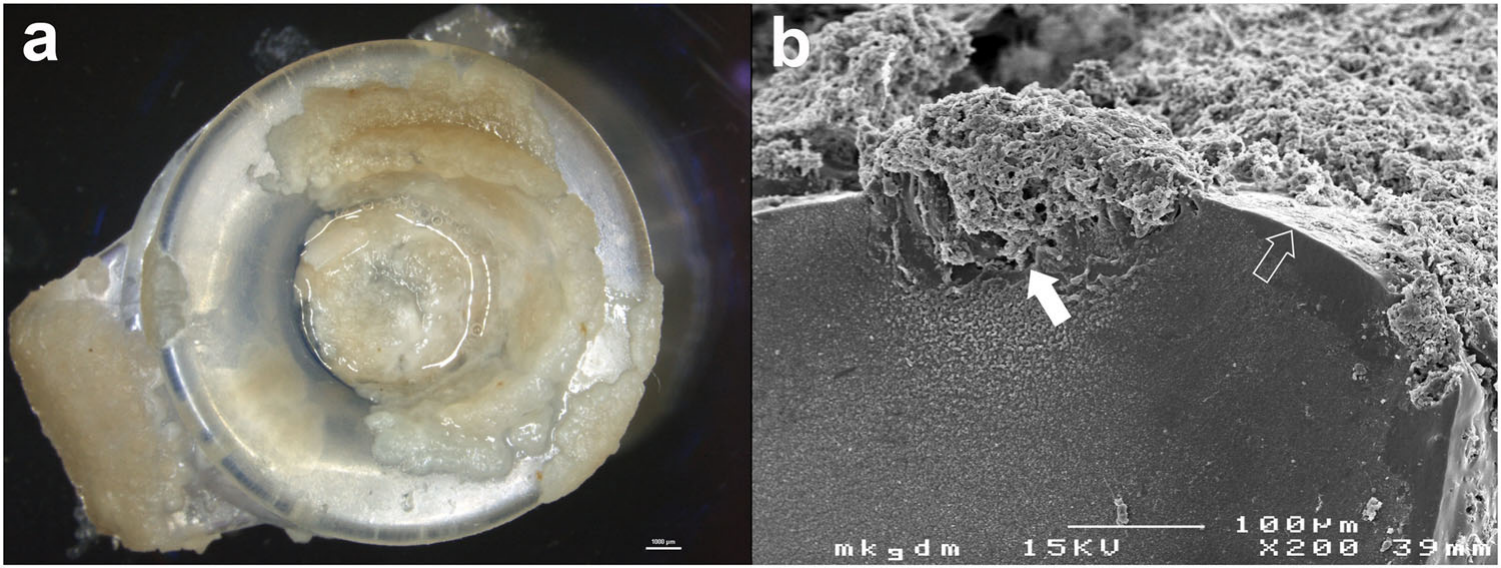
**a** View of a dysfunctional Provox 2 voice prosthesis at explantation after 96 days in situ. The valve flap and esophageal flange are overgrown by candida based mixed biofilm deposits. Transprosthetic leakage of esophageal contents is caused by impairment of the valve closure due to biofilm deposits and deterioration of the silicone material. **b** Candida species infiltrate the silicone material of a VP after weeks (cross section of a Provox 2 after 48 weeks in situ, white arrow: site of biofilm infiltration, shaped arrow: surface of the silicone material)

Microbial colonization of medical polymer devices and implants cannot be avoided, if used in non-sterile body compartments. For example, in otorhinolaryngology, voice prostheses (VPs) for secondary voice rehabilitation in laryngectomized patients fail to function after only months in situ because of biofilm formation [1]. VPs are inserted in a tracheoesophageal fistula between trachea and esophagus and allow the passage of air from the airways into the neopharynx, where mucosal folds produce a sound by airstream induced vibration. However, during swallowing the valve mechanism must remain closed to keep nutrition and fluids constrained to the esophagus and to prevent aspiration and pneumonia. These prostheses are constantly exposed to the oropharyngeal microbiome that comprises several hundred resident bacterial and fungal strains. These commensals mainly include viridans streptococci, staphylococci and especially candida species. They interact synergistically and form microbial biofilms that have been documented to regularly cause failures of VPs in laryngectomized patients [2–4]. The multispecies biofilms grow mainly on the esophageal valve parts of the VPs (flange and valve flap) and can be inspected after explantation as thick covers of biofilm that impair the valve closure (Fig.1a). Recent clinical studies have shown a decreasing device lifetime of modern VPs from 3–5 to approximately 2 months [5]. This puts emphasis on the demand for more durable prosthetic materials, antibiofilm coatings or bioactive surfaces for VPs [6, 7]. Interestingly, among the variety of static and dynamic microtiter plate assays, disc models and flow models, there is no consensus on in vitro testing procedures, microbial compositions, incubation times and even growth media to simulate this multispecies biofilm formation on VPs in the tracheoesophageal environment. The “artificial throat”, a device specifically adapted to in vitro examination of VPs was introduced by Leunisse et al. and has been used in several studies as modified Robbins device, with varying culturing parameters [8–13]. Other studies have adapted their testing protocols from single species biofilms or have used commonly recommended growth media, such as yeast peptone dextrose medium (YPD), RPMI 1640, yeast nitrogen base medium (YNB) or tryptic soy broth (TSB), without prior evaluation for a specific multispecies composition [14]. Also, recommendations of fetal bovine serum (FBS) or spider medium can be found to specifically stimulate hypheal growth of Candida species, which is considered to be the more infiltrative growth form. Only little data on optimum growth media for specific fungal-bacterial compositions, such as oropharyngeal biofilms, is available [15]. This study comprises an in-vitro model, that aims at the generation of an oropharyngeal biofilm composition on silicone for testing of antibiofilm materials or coatings for VPs using non-invasive evaluation of growth kinetics over weeks. In a first step, an optimum growth medium needs to be identified to define a standard biofilm growth curve for medical grade silicone in this model. As the usually mixed microbial compositions on VPs comprise Candida albicans, Candida tropicalis, Streptococcus salivarius, Streptococcus epidermidis, Staphylococcus aureus, Rothia dentocariosa and Lactobacillus gasseri, the possible benefit of combining growth media (mixtures of YPD, FBS, RPMI 1640 and brain heart infusion (BHI) is investigated to enhance longterm growth of all combined species as one polymicrobial biofilm. The macroscopic biofilm growth, the microscopic structure, and the presence of fungal hyphae as precondition for infiltration into the silicone material, were assessed by 2D image analysis of living biofilm depsits. After completion of a 22 day cultivation period, scanning electron microscopy (SEM) of the biofilm deposits was performed for all growth media in either single and combined application.

## Materials and methods

### Preparation of microbial strains

Microbial strains of C. albicans, C. tropicalis, S. aureus, S. epidermis and S. salivarius originated from a collection of explanted dysfunctional voice prostheses of laryngectomized patients visiting the Department of Phoniatrics-Logopedics of the Medical University Hospital. VPs with macroscopic biofilm infestation were withdrawn and sonicated in phosphate buffered saline solution (PBS, Morphisto, Frankfurt am Main, Germany) for 10 min to remove lose biofilm debris and vortexed in 5 ml PBS for 3 min before the specimen were isolated and identified on agar plates using standard microbiological methods. Bacterial strains for composition of the in-vitro biofilm were selected based on their frequent appearance on explanted prostheses and on reports in literature. Isolated R. dentocariosa and L. gasseri strains were provided from a collection by the Department of Microbiology of the Medical University Hospital. All specimens were stored at −80 °C and thawed before further use.

### Preparation of silicone material samples

Platelets of 8 mm diameter and 1 mm thickness were punched out of blue colored medical grade silicone sheets (Websinger, Wolkersdorf, Austria) and a segment was cut off to mark the bottom side of each platelet. The platelets were mounted on surgical steel tips for incubation in a vertical position to avoid settlement of planktonic cells by gravity. The prepared samples were autoclaved for 20 min at 125 °C and placed sterile in well titer plates (CellStar Greiner bio-one, Kremsmünster, Austria). In each of the following growth media, mixed biofilms were grown on 12 platelets.

### Preparation of growth media

Following growth media were selected:

- YPD (Yeast extract peptone dextrose: yeast extract 1% (Sigma-Aldrich Life Science, St. Louis, USA), glucose 2% (Merck KGaA, Darmstadt, Germany), peptone water 2% (Oxoid LTD, Hamshire, England)) and FBS (Fetal Bovine Serum: (Gibco, Life Technologies Carlsbad, California, USA)) were used as control growth media and to prepare the following mixtures:
- BHI 30% (Sigma-Aldrich life science, St. Louis, USA) + YPD 70%
- FBS 30% + YPD 70%
- FBS 30% + RPMI 70% medium + 2% glucose (RPMI 1640: 20,8 g RPMI-1640 (Sigma-Aldrich life science, St. Louis, USA), 69,06 g MOPS (Sigma-Aldrich life science, St. Louis, USA) 36 g glucose (Merck KGaA, Darmstadt, Germany)
- FBS 30% + YNB 70% (Yeast nitrogen base 0,67% with ammonium sulphate without dextrose or amino acids (Sigma-Aldrich life science, St. Louis, USA), glucose 2% (Merck KGaA, Darmstadt, Germany)
- YPD 50% + RPMI 1640 50%

### Preparation of inoculum

The frozen candida strains were inoculated with sterile loops, plated out on Sabouraud-Dextrose agar (Becton Dickinson, New Jersey, USA) and Columbia 5% sheep blood agar (bioMerieux SA, Marcy l’Etoile, France) and incubated at 37 °C for 24 h. Single colonies of each candida species were picked from the Sabouraud-Dextrose agar, inoculated in each 20 ml of YPD and then incubated on an orbital shaker at 100 rpm for 24 h. The overnight candida cultures were centrifuged for 5 min and the supernatants discarded. The remaining cells were washed three times with PBS. The washed planktonic candida cells were used to prepare 1.0 McFarland standard (equaling 10 × 10–6 cfu/ml), inoculates in distilled water, which were then mixed into one microbial suspension. S. aureus, S. epidermidis and S. salivarius were suspended in PBS to a cell density of 10 × 6 cfu/ml and then added to the C. albicans suspension. One milliliter of each suspension was mixed into the final suspension and then a 10 fold dilution was performed.

### Biofilm growth

All silicon platelets were pre-coated with bovine serum for 24 h at 37 °C to improve initial microbial adhesion capability. Then one milliliter of the prepared microbial suspension was added to 9 ml of each growth medium. The growth media were then added to the silicone platelets in the micro titer plates. After 24 h of incubation at 37 °C at 150 rpm, the growth media were removed from the well plates and replenished with fresh growth media and planktonic cells. This reseeding procedure was performed daily for 22 days.

### Biofilm analysis

**Fig. 2:**
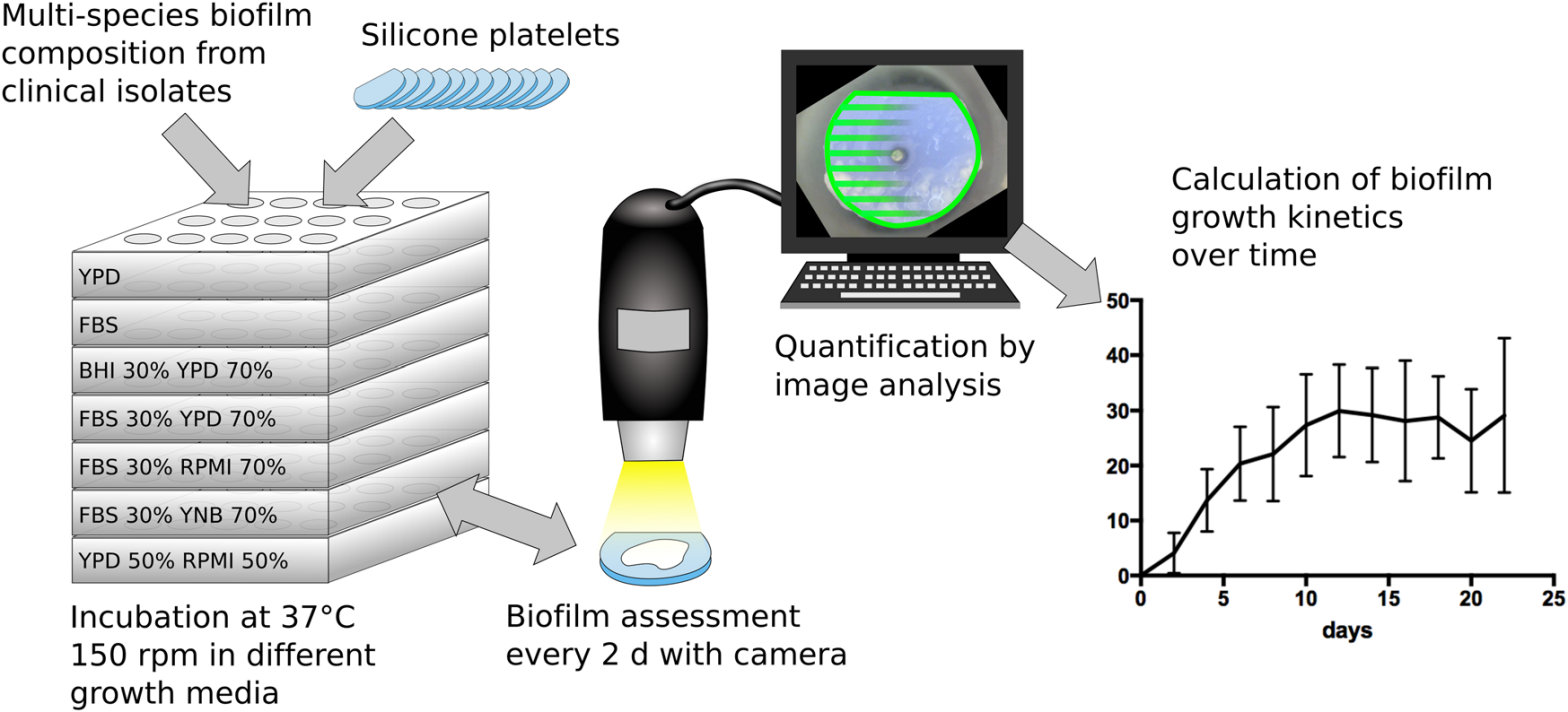
Scheme of the applied microtiter biofilm model and quantification of macroscopic size of biofilm deposits using image analysis software. The size of biofilm cover was calculated as percentage of each total platelet surface. (Artwork created with Inkscape 0.91)

The applied in vitro biofilm model and the quantification of biofilm deposits are illustrated in Fig. 2. Every two days the platelets were washed gently with sterile PBS and photographed top down at constant distance and lighting to size and distribution of biofilm deposits. The sizes of the biofilm deposits were calculated using the Biofilm Cartographer software introduced by Leonhard et al. (Version 2.9, commercially not available, Medical University, Vienna, Austria) [16]. The surface covered by biofilm was assessed as percentage of the total platelet surface (100%). After 22 days the assessed biofilm distributions on each series of platelets were digitally merged into color coded images to visualize areas of permanent biofilm coverage over the course of time. All platelets were rinsed with PBS and the biofilms were fixed with 2.5% glutaraldehyde at 4 °C for 24 h. The samples were dehydrated by immersion in a series of ethanol solutions ranging from 70% (v/v) ethanol in distilled water to absolute ethanol, then chemically dried with Hexamethyldisilazane (HMDS, Sigma-Aldrich Life Science, Lt. Louis, USA) and sputtered with gold (Sputter Coater: SC502, Polaron, Fisons Instruments, Surface Science Division, Cambridge, UK). Microscopic structures of biofilm deposits on four representative platelets per growth medium were further analyzed with SEM (JSM 6310, JEOL Ltd., Tokyo, Japan).

### Statistical analysis

Statistical calculation of the growth of biofilm deposits was performed using IBM SPSS Statistics (version 23, IBM Corporation, New York, United States) and graphs were illustrated using Graphpad Prism software (Version 7.0a, GraphPad Software, Inc., La Jolla, CA 92037 USA). The mean value of 12 platelets was calculated every two days for each growth medium. Percentages of the covered areas were arcsine transformed in order to remove the correlation between mean and standard deviation. Transformed data were analyzed by a general estimation equation model with an also autoregressive correlation structure and material as a group factor and day of measurement as within group factor. Comparison between the growth media were done by Bonferroni sequential tests. For all comparisons, a p-value < 0.05 was chosen as significance level.

## Results

### Microscopic biofilm growth

**Fig. 3:**
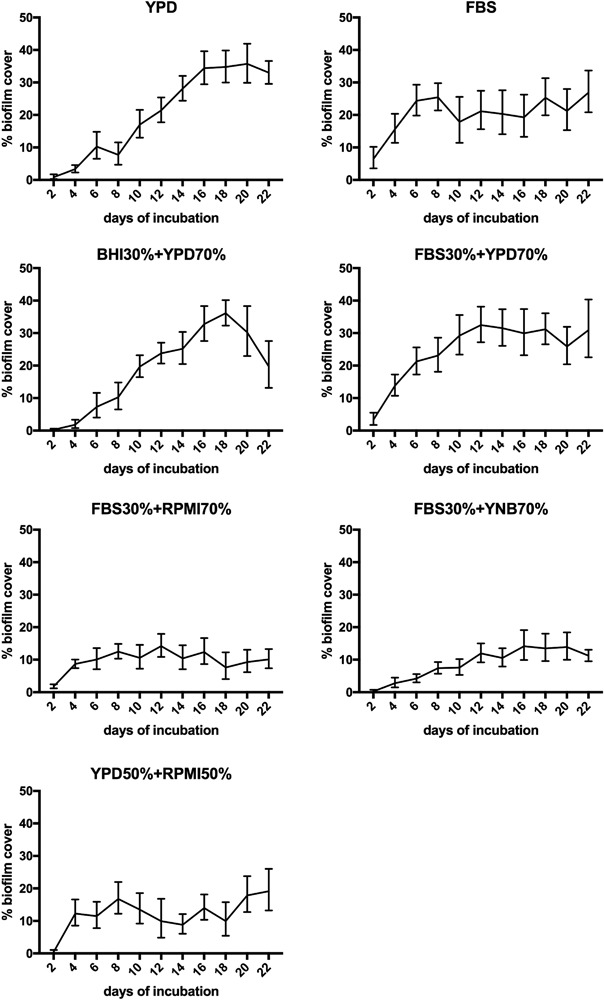
Overview of growth kinetics of biofilm deposits over 22 days in the tested growth media: yeast peptone dextrose (YPD), fetal bovine serum (FBS) and mixtures with brain heart infusion (BHI), yeast nitrogen base (YNB) and RPMI 1640 (RPMI)

Macroscopic biofilm growth was first detected on all test platelets latest after 4 days and continued over the following 22 days. For each tested growth medium, growth kinetics of the same biofilm composition over the course of time is illustrated as diagrams in Fig. 3. YPD and FBS served as controls and the biofilm growth showed no significant statistic difference between both growth media (p > 0.43). YPD generated a maximum total biofilm cover of > 30% after 16 days of incubation, which was about 10% above the maximum achieved with FBS, but the onset of visible macroscopic growth was earlier with FBS (day 2: p = 0.053, day 4–8: p < 0.05). Interestingly, the mixture of FBS 30% + YPD 70% seemed to synergistically blend the effects of a rapid onset and a higher total biofilm cover together. Compared to BHI 30% + YPD 70%, more biofilm mass was assessed in the first 6 days of incubation (day 2–6: p < 0.05), but after the first week no significant difference in the performance could be found. In contrast, mixtures containing RPMI 1640 and YNB medium produced significantly less visible total biofilm mass over the whole observation period.

### Microscopic biofilm morphologies

**Fig. 4:**
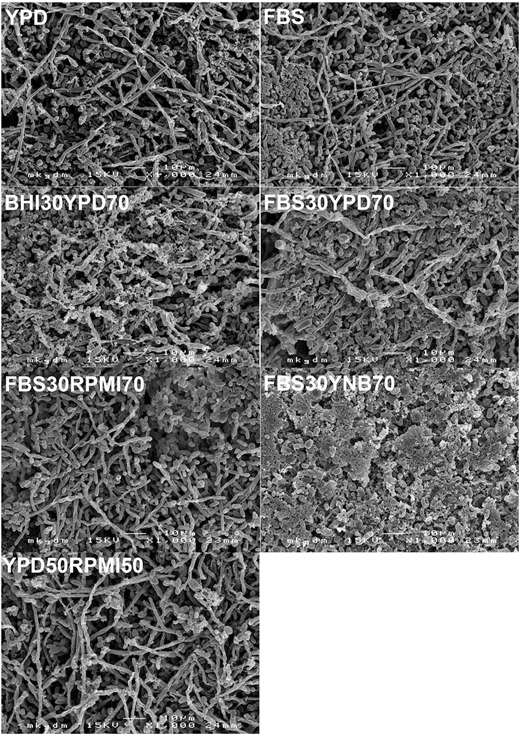
Scanning electron micrographs of the resulting biofilm structures assessed on silicone after 22 days in the in vitro biofilm model. Key structures of biofilms on explanted voice prostheses, such as budded yeast, hypheal germination and balanced bacterial proliferation were assessed in all growth media tested, except in the mixture of FBS 30% + YNB 70%

The microscopic analysis focused on the presence of cell morphologies (hyphae and budded yeast cells) and the distribution of species between bacteria (cocci and rod shaped bacilli) and fungi. The collapsed extracellular matrix could not be evaluated due to shrinking during dehydration. Overall, the microscopic configuration of biofilm deposits on all platelets was very heterogeneous, displaying nests of staphylococci, chains of streptococci and budded yeasts side by side with areas of dense hypheal proliferation with varying tube lengths. Rich proliferation of staphylococci, streptococci and budded yeast forms was supported by all growth media. The presence of hyphae was assessed most frequently in FBS, YPD and FBS 30% + YPD 70%. Notably, rod shaped bacilli were found regularly in BHI 30% + YPD 70%, FBS 30% + YPD 70% and YPD, while none were assessed in the mixtures of FBS 30% + RPMI 70% and FBS 30% + YNB 70%. An overview of the resulting biofilm morphologies for all growth media is displayed in Fig. 4.

### Distribution of macroscopic biofilm deposits

Using the mapping function of the image analysis software, the areas of more permanent biofilm aggregation (red colors) can be distinguished from areas with less biofilm aggregation (green colors) over time on each platelet (see electronic supplementary material 1). It shows that the top edges of the platelets are areas of frequent and permanent colonization, which can be explained by the physical effect, that particles/cells tend to agglomerate near the surface of agitated fluids. The largest areas of permanent biofilm colonization were detected in FBS 30% + YPD 70%, BHI 30% + YPD 70%, FBS and YPD.

### Discussion

Multispecies biofilm formation by bacteria and fungi is a clinical problem of indwelling medical devices, if used in non sterile body compartments. They need to be replaced frequently due to biofilm induced loss of function [17, 18]. Silicone is the material of choice for modern standard VPs due to its biocompatibility and flexibility, which allows folding and atraumatic replacement [19]. Since microbial colonization of the silicone material in VPs cannot be avoided due to the exposed location in the esophagus, protective strategies such as resistant materials, inhibitive coatings or repellent surfaces are needed to improve the device lifetime of standard VPs. Initial evaluation of such strategies using rapid and cost-effective in-vitro biofilm models is an essential step, before producing prosthesis prototypes, that meet the quality requirements of implantable products and can be studied in-vivo.

Biofilms on VPs typically comprise several cross-kingdom interacting communities of bacteria and fungi that evolve into valve blocking deposits and degrade/infiltrate the silicone after weeks (Fig. 1b) [20]. The applied microbial composition has been selected from explanted voice prostheses of patients with a history of repeatedly short device lifetimes due to biofilm associated valve leakage or material infiltration and is consistent with existing literature on in-vivo colonization of VPs [3, 4]. To remain as close as possible to in-vivo conditions, microbial isolates from patients, who have repeatedly presented excessive biofilm infestation and aggressively infiltrated voice prostheses in-vivo, were used in this study. Pre-evaluation showed no difference in biofilm formation in-vitro between ATCC strains and the used strains. While strong biofilm formation is categorized in ATCC strains, to our knowledge, the ability of silicone material infiltration is not, but it is important for future testing of microbial degradation of prosthetic materials. Active interactions between C. albicans, oral streptococci and S. aureus have been described, though it is not fully understood, which co-factors lead to synergistic or antagonistic coexistence inside a biofilm [21–23]. R. dentocariosa and lactobacilli are discussed to play key roles in co-adhesion and transition to hypheal growth of C. albicans, and seem to be associated with early device failure [24, 25]. Using mixtures of standard growth media to support the microbial growth of all these species is plausible, but only limited data on in-vitro cultivation of oropharyngeal biofilms on silicone or VPs exists. A mixture of 30% BHI and 70% of a defined yeast medium has been used in a Modified Robins Device to illustrate the protective effect of probiotics, antibiofilm coatings or effects of co-incubation of candida with bacterial strains [10, 26, 27]. Wannemuehler et. al. investigated a vibratory stimulus on biofilms on VPs using a 1:1 mixture of BHI and YPD. In a previous study by our group, a two species biofilm of S. salivarius and C. albicans was incubated in RPMI 1640 on medical grade silicone for 140 days [16]. However, such variant incubation protocols are a known problem for comparing the results of in-vitro simulations and the efficacy of antibiofilm measures [28]. Simulation of complex multispecies formulations, such as oropharyngeal biofilms, in in-vitro models should be preceded by a pre-evaluation of growth media to optimally support growth of stable biofilm deposits, structures and cell morphologies and to achieve similar to in-vivo findings including a balanced proliferation of all involved species over a period of time that is representative for the clinical application of a particular implant or prosthesis. In the present model, initial evaluation of commonly applied single growth media, that have been recommended for the specific species or have been used in similar models, confirmed YPD and FBS as eligible in regard to biofilm mass, stability and cell growth forms, but differences in longterm performance and support of all species between the tested growth media have been noticed (data not published). Goal of this study was therefore to investigate, if combinations of these growth media could be used to further optimize longterm biofilm growth of oropharyngeal biofilm compositions on silicone.

The results show, that YPD, FBS and mixtures of FBS 30% + YPD 70% or BHI 30% + YPD 70% are eligible to generate long lasting oropharyngeal-like biofilm compositions on medical grade silicone for testing purposes. The mixture of YPD and FBS shows a synergy of accelerated onset of growth with the maximum achieved macroscopic biofilm cover. Notably for this longterm biofilm model, addition of RPMI and YNB to a mixture produced significantly less macroscopically visible biofilm mass. Further, the addition of RPMI or YNB to FBS showed no growth support of rod shaped lactobacilli in SEM analysis, whereas they were frequently identified in the other mixtures, including YPD 50% + RPMI 50%. Analysis of key morphological structures showed that all growth media except FBS 30% + YNB 70% produced solid macroscopic multispecies biofilms that are similar to findings on dysfunctional voice prostheses. In FBS 30% + YNB 70%, hypheal growth appeared to be reduced and an increased mass of bacteria could be identified. However, the platelets displayed abundant local morphological heterogeneity, which can be interpreted as a previously described organization in functional micro-consortia [29].

## Conclusion

Growth media have impact on in-vitro formation of biofilms and need pre-evaluation to be adjusted for specific polymicrobial compositions. Mixtures of FBS 30% + YPD 70% and BHI 30% + YPD 70% proved to produce stable oropharyngeal-like biofilm deposits over weeks, and show similar microscopic cell morphologies to in-vivo findings of VPs. Application of these growth media mixtures in the presented biofilm model can be used to screen novel polymer materials, coatings or surface modifications that are intended to inhibit or slow biofilm formation on future VP designs.

## Electronic supplementary material

Supplementary Figure
