# Supplementary figures and images for: Evaluation of combined growth media for in vitro cultivation of oropharyngeal biofilms on prosthetic silicone
Source: J Mater Sci Mater Med. 2018 Apr 9;29(4):45. doi: 10.1007/s10856-018-6051-7 (PMC5891558; doi:10.1007/s10856-018-6051-7)

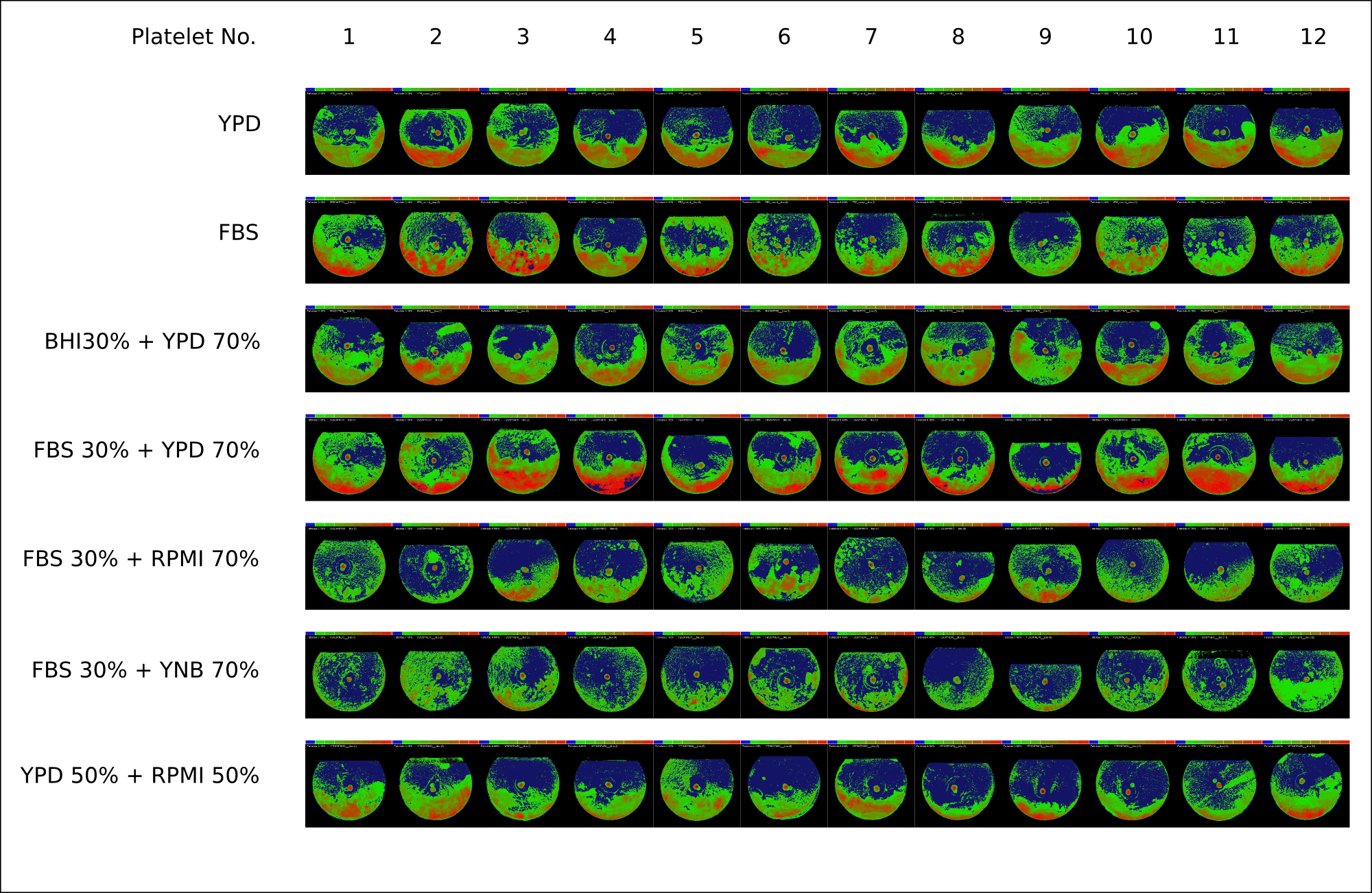

Supplement: Supplementary file 1 — Supplementary Figure [file 10856_2018_6051_MOESM1_ESM.jpg]
